# Divergent roles for antigenic drive in the aetiology of primary versus dasatinib-associated CD8^+^ TCR-Vβ^+^ expansions

**DOI:** 10.1038/s41598-017-18062-x

**Published:** 2018-02-07

**Authors:** Anna Lissina, James E. McLaren, Mette Ilander, Emma I. Andersson, Catherine S. Lewis, Mathew Clement, Andrew Herman, Kristin Ladell, Sian Llewellyn-Lacey, Kelly L. Miners, Emma Gostick, J. Joseph Melenhorst, A. John Barrett, David A. Price, Satu Mustjoki, Linda Wooldridge

**Affiliations:** 1 Faculty of Health Sciences, University of Bristol, Biomedical Sciences Building, Bristol, UK; 2 Institute of Infection and Immunity, Cardiff University School of Medicine, Heath Park, Cardiff, UK; 3 Hematology Research Unit Helsinki, Department of Clinical Chemistry and Hematology, University of Helsinki and Helsinki University Hospital Comprehensive Cancer Center, Helsinki, Finland; 4 Stem Cell Allogeneic Transplantation Section, Hematology Branch, National Heart, Lung, and Blood Institute, National Institutes of Health, Bethesda, Maryland USA

## Abstract

CD8^+^ T-cell expansions are the primary manifestation of T-cell large granular lymphocytic leukemia (T-LGLL), which is frequently accompanied by neutropenia and rheumatoid arthritis, and also occur as a secondary phenomenon in leukemia patients treated with dasatinib, notably in association with various drug-induced side-effects. However, the mechanisms that underlie the genesis and maintenance of expanded CD8^+^ T-cell receptor (TCR)-Vβ^+^ populations in these patient groups have yet to be fully defined. In this study, we performed a comprehensive phenotypic and clonotypic assessment of expanded (TCR-Vβ^+^) and residual (TCR-Vβ^−^) CD8^+^ T-cell populations in T-LGLL and dasatinib-treated chronic myelogenous leukemia (CML) patients. The dominant CD8^+^ TCR-Vβ^+^ expansions in T-LGLL patients were largely monoclonal and highly differentiated, whereas the dominant CD8^+^ TCR-Vβ^+^ expansions in dasatinib-treated CML patients were oligoclonal or polyclonal, and displayed a broad range of memory phenotypes. These contrasting features suggest divergent roles for antigenic drive in the immunopathogenesis of primary versus dasatinib-associated CD8^+^ TCR-Vβ^+^ expansions.

## Introduction

T-cell large granular lymphocytic leukemia (T-LGLL) is a chronic lymphoproliferative disorder characterized by the clonal expansion of mature CD3^+^ CD8^+^ cells^1–5^. Although the incidence of T-LGLL is relatively low, it nonetheless occurs more commonly than other proliferative aberrations within the CD8^+^ T-cell compartment, and there is no effective cure^6^. The disease typically afflicts individuals later in life (mean age of onset, ~60 years), but can also develop after allogeneic organ or stem cell transplantation^4,7^. Neutropenia complicates 70–80% of cases^4,8,9^. In addition, T-LGLL is strongly associated with autoimmune disorders, most commonly rheumatoid arthritis (RA), which affects ~30% of patients^4,10^. T-LGLL is currently managed with low-dose immunosuppressive agents^6^, primarily to combat the clinical manifestations of neutropenia, but response rates remain suboptimal. A better understanding of the condition is therefore required to guide novel and more specific therapeutic interventions.

CD8^+^ T-cell expansions have also been reported in patients undergoing treatment with the promiscuous tyrosine kinase inhibitor dasatinib, which is licensed as a first line therapeutic option in the management of chronic myelogenous leukemia (CML) and Philadelphia chromosome-positive (Ph^+^) acute lymphoblastic leukemia (ALL)^11–16^. In some cases, these expanded CD8^+^ T-cells can even mimic T-cell large granular lymphocytes (T-LGLs). Dasatinib-associated CD8^+^ T-cell expansions have been linked with adverse side-effects, including pleural effusions and colitis^14,15^, and beneficial outcomes, including delayed progression and long-term remission in leukemia patients^12,13,17^.

A number of studies have suggested that clonal CD8^+^ T-cell expansions in T-LGLL patients either arise in response to an unknown persistent antigen^18,19^ or occur via neoplastic transformation of genes involved in cellular homeostasis or proliferation^20–22^. An alternative view is that such expansions originate within a primary antigen-specific response and then acquire genetic mutations that confer additional proliferative and/or survival advantages^1^. Particular attention has been devoted in this regard to the Janus kinase (JAK)/signal transducer and activator of transcription (STAT) pathway^23^. Indeed, a large proportion of T-LGLL patients have been found to harbor somatic gain-of-function mutations in genes encoding the STAT family of proteins^24^. The majority of these mutations affect *STAT3*^25–27^, with others localizing most commonly to *STAT5B*^28^.

To inform these distinct mechanistic hypotheses, we performed an in-depth comparison of CD8^+^ T-cell expansions in T-LGLL and dasatinib-treated CML patients. A molecular approach was used to identify T-cell receptor (TCR) clonotypes within the expanded TCR-Vβ-defined CD8^+^ T-cell populations, and flow cytometry was used to assess multiple phenotypic markers with reference to non-expanded (TCR-Vβ^−^) and cytomegalovirus (CMV)-specific CD8^+^ T-cells. The data reveal striking phenotypic and clonotypic differences that suggest divergent roles for antigenic drive in the aetiology of primary versus dasatinib-associated CD8^+^ TCR-Vβ^+^ expansions.

## Results

### Striking differences in the size and differentiation status of dominant CD8^+^ TCR-Vβ^+^ expansions in T-LGLL and dasatinib-treated CML patients

**Figure 1:**
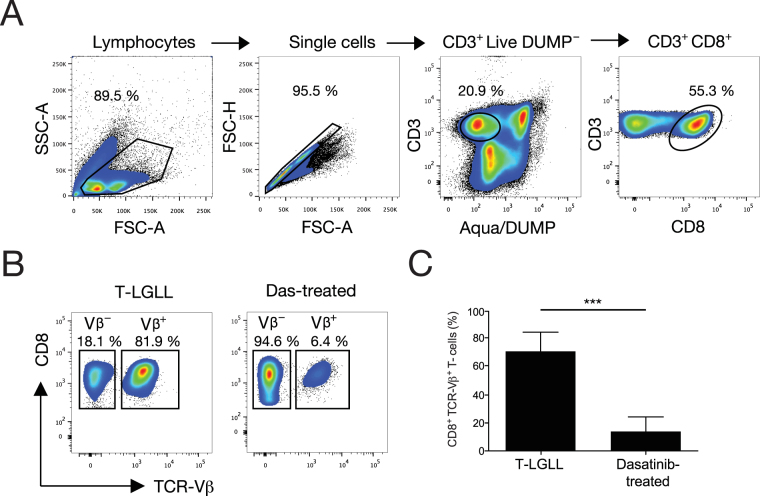
Quantification of dominant CD8^+^ TCR-Vβ^+^ expansions in T-LGLL and dasatinib-treated CML patients. (**A**) Gating strategy for the identification of viable CD14^−^ CD19^−^ CD3^+^ CD8^+^ cells within PBMCs. (**B**) Representative flow cytometry plots showing expanded (TCR-Vβ^+^) and residual (TCR-Vβ^−^) CD8^+^ T-cell populations in T-LGLL and dasatinib-treated CML patients. (**C**) Percent frequencies of dominant CD8^+^ TCR-Vβ^+^ expansions in T-LGLL (n = 11) and dasatinib-treated CML patients (n = 10). Statistical comparisons were performed using the Mann-Whitney U test. ***P < 0.0001. Error bars represent SD.

**Figure 2:**
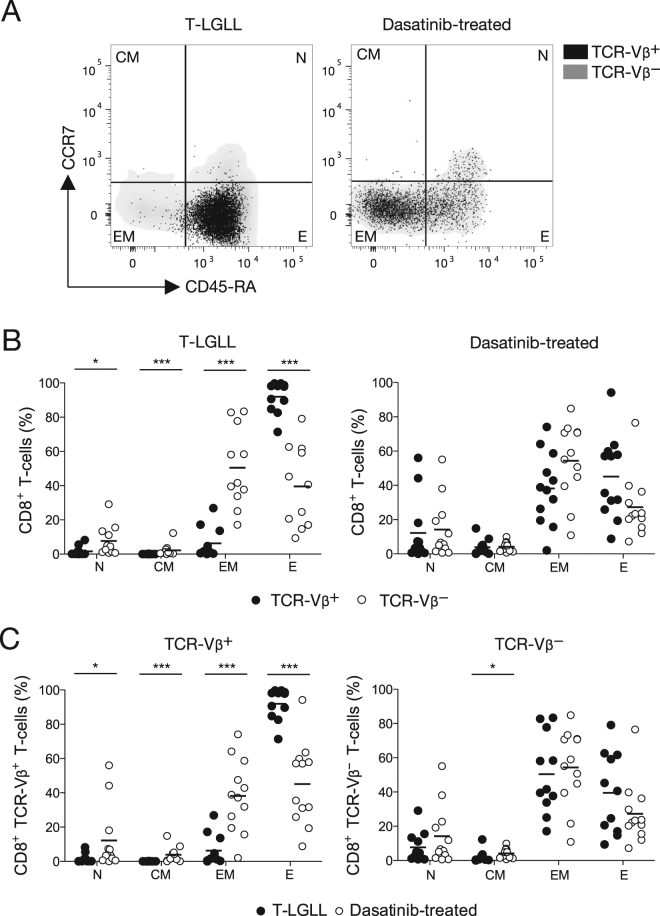
Differentiation status of expanded (TCR-Vβ^+^) and residual (TCR-Vβ^−^) CD8^+^ T-cells in T-LGLL and dasatinib-treated CML patients. (**A**) Representative flow cytometry plots showing CCR7 and CD45-RA expression among TCR-Vβ^+^ (black dots) and TCR-Vβ^−^ CD8^+^ T-cells (grey density clouds) in T-LGLL (left panel) and dasatinib-treated CML patients (right panel). (**B**) Percent frequencies of TCR-Vβ^+^ (black dots) and TCR-Vβ^−^ CD8^+^ T-cells (white dots) displaying the indicated differentiation phenotypes in T-LGLL (left panel) and dasatinib-treated CML patients (right panel). (**C**) Percent frequencies of TCR-Vβ^+^ (left panel) and TCR-Vβ^−^ CD8^+^ T-cells (right panel) displaying the indicated differentiation phenotypes in T-LGLL (black dots) and dasatinib-treated CML patients (white dots). Statistical comparisons were performed using the Mann-Whitney U test. ***P < 0.0001, *P < 0.01. Horizontal bars indicate mean values. N, naïve; CM, central-memory; EM, effector-memory; E, effector.

Peripheral blood mononuclear cell (PBMC) samples from T-LGLL and dasatinib-treated CML patients (Table 1) were characterized by flow cytometry (Fig. 1A,B). Dominant TCR-Vβ^+^ expansions were identified using a panel of monoclonal antibodies (mAbs) covering 75% of the *TRBV* gene-encoded repertoire, and phenotypically distinct subsets of CD8^+^ T-cells were classified as follows: naïve (N, CCR7^+^ CD45-RA^+^); central-memory (CM, CCR7^+^ CD45-RA^−^); effector-memory (EM, CCR7^−^ CD45-RA^−^); and effector (E, CCR7^−^ CD45-RA^+^). The dominant TCR-Vβ^+^ expansions in T-LGLL patients were significantly larger than the dominant TCR-Vβ^+^ expansions in dasatinib-treated CML patients (Fig. 1B,C, Supplementary Figure 1, Table 1). Moreover, the dominant TCR-Vβ^+^ expansions in T-LGLL patients were almost exclusively populated with terminally differentiated (CCR7^−^ CD45-RA^+^) effector CD8^+^ T-cells, whereas the dominant TCR-Vβ^+^ expansions in dasatinib-treated CML patients were more broadly constituted across the phenotypic spectrum of CD8^+^ T-cells (Fig. 2A).

**Table 1:** Clinical details of T-LGLL and dasatinib-treated CML patients.

Patient ID	Leukemia type	Additional clinical complications	Age at diagnosis (years)	Age at sample collection (years)	Duration of dasatinib treatment at sample collection (years, months)	Sex	Dominant TCR-Vβ expansion (Arden nomenclature)	Size of TCR-Vβ expansion (% of CD8^+^ T-cell population)	Presence of STAT3 or STAT5B mutations
1	T-LGLL	Neutropenia, thrombocytopenia, leukopenia, B-cell dyscrasia, hypergammaglobulinemia	58	64	N/A	F	1	51.1	STAT3
2	T-LGLL	B-cell dyscrasia, unspecified collagenosis	72	73	N/A	F	22	81.9	STAT5B
3	T-LGLL	Anemia, neutropenia, seronegative RA	74	74	N/A	M	16	66.1	STAT3
4	T-LGLL	Anemia, neutropenia, thrombocytopenia	66	67	N/A	M	5.1	72	No
5	T-LGLL	None	59	59	N/A	M	3	94.3	No
6	T-LGLL	None	50	50	N/A	F	17	82.8	STAT5B
7	T-LGLL	Neutropenia, anemia	27	38	N/A	M	20	73.4	STAT3
8	T-LGLL	Neutropenia	24	37	N/A	F	14	70.5	STAT3
9	T-LGLL	Neutropenia	50	56	N/A	F	13.6	84	STAT3
10	T-LGLL	Anemia, thrombocytopenia	85	87	N/A	M	8	62.5	No
11	T-LGLL	Anemia	65	67	N/A	M	2	57	No
12	CML	None	71	72	1, 7	F	13.2	43.4	No
13	CML	None	44	44	0, 6	F	14	7.25	No
14	CML	None	60	62	1, 2	F	3	9.67	No
15	CML	Ulcerative colitis	47	47	0, 6	M	3	10.7	No
16	CML	None	28	45	4, 7	M	5.3	5.61	No
17	CML	None	45	46	1, 2	M	7.2	31	No
18	CML	Pleural effusion	58	59	0, 8	M	2	8.8	No
							8	9.99	
19	CML	None	67	70	2, 4	M	22	11.9	No
							23	13.1	
20	CML	None	45	46	0, 9	F	2	12.4	No
21	CML	None	58	65	1, 1	F	14	15.5	No

Side-by-side comparisons of the expanded (TCR-Vβ^+^) and residual (TCR-Vβ^−^) CD8^+^ T-cell populations revealed an inverted EM/E profile in T-LGLL patients. Terminally differentiated effector cells predominated in the TCR-Vβ^+^ fraction (mean, ~90%), but were significantly less common in the TCR-Vβ^−^ fraction (mean, ~40%), which incorporated higher frequencies of naïve and less differentiated memory cells (Fig. 2B, left panel). In contrast, the expanded (TCR-Vβ^+^) and residual (TCR-Vβ^−^) CD8^+^ T-cell populations were phenotypically alike in dasatinib-treated CML patients, with a relatively balanced EM/E profile reflecting mean frequencies of ~40% for each subset in the TCR-Vβ^+^ fraction (Fig. 2B, right panel). Accordingly, the dominant CD8^+^ TCR-Vβ^+^ expansions in T-LGLL patients were significantly more differentiated than the dominant CD8^+^ TCR-Vβ^+^ expansions in dasatinib-treated CML patients (Fig. 2C, left panel).

The residual (TCR-Vβ^−^) CD8^+^ T-cell populations were phenotypically similar in T-LGLL and dasatinib-treated CML patients, mirroring the subset distribution normally observed in healthy donor PBMCs (Fig. 2C, right panel). However, relatively low frequencies of naïve and central-memory cells were present in both groups, potentially reflecting advanced age in the T-LGLL cohort (mean, 61 years) and preferential expansion of effector-memory cells in dasatinib-treated CML patients. Of note, considerable variability was detected among patients with respect to the frequency of each subset in the TCR-Vβ^−^ fraction, and a comparably broad inter-individual spread was observed among dasatinib-treated CML patients in the TCR-Vβ^+^ fraction (SD: N, 17.2; CM, 4; EM, 23.6; E, 24.4). In contrast, subset distribution was tightly clustered among T-LGLL patients in the TCR-Vβ^+^ fraction (SD: N, 2.7; CM, 0.07; EM, 8.9; E, 9.2).

### Dominant CD8^+^ TCR-Vβ^+^ expansions in T-LGLL and dasatinib-treated CML patients are phenotypically distinct

**Figure 3:**
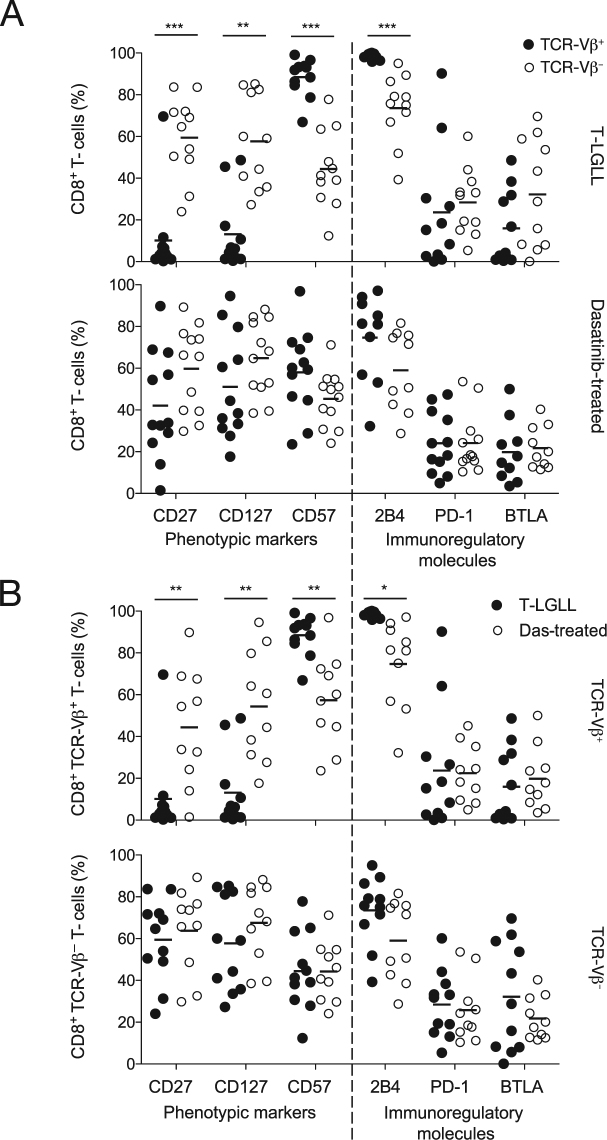
Phenotypic analysis of expanded (TCR-Vβ^+^) and residual (TCR-Vβ^−^) CD8^+^ T-cells in T-LGLL and dasatinib-treated CML patients. (**A**) Percent frequencies of TCR-Vβ^+^ (black dots) and TCR-Vβ^−^ CD8^+^ T-cells (white dots) expressing the indicated markers in T-LGLL (top panel) and dasatinib-treated CML patients (bottom panel). (**B**) Percent frequencies of TCR-Vβ^+^ (top panel) and TCR-Vβ^−^ CD8^+^ T-cells (bottom panel) expressing the indicated markers in T-LGLL (black dots) and dasatinib-treated CML patients (white dots). Statistical comparisons were performed using the Mann-Whitney U test. ***P < 0.0001, **P < 0.001, *P < 0.01. Horizontal bars indicate mean values.

In T-LGLL patients, the dominant CD8^+^ TCR-Vβ^+^ expansions displayed a classical antigen-driven phenotype, reflected by significantly higher expression levels of CD57 and 2B4 and significantly lower expression levels of CD27 and CD127 compared with the residual CD8^+^ TCR-Vβ^−^ populations (Fig. 3A, top panel). The dominant CD8^+^ TCR-Vβ^+^ expansions in these patients were also characterized by a lack of CTLA-4, CD160, and Tim-3 (data not shown), and relatively low expression levels of PD-1 and BTLA (Fig. 3A, top panel). In contrast, there were no significant phenotypic differences between the expanded (TCR-Vβ^+^) and residual (TCR-Vβ^−^) CD8^+^ T-cell populations in dasatinib-treated CML patients (Fig. 3A, bottom panel).

Inter-group comparisons revealed that the dominant CD8^+^ TCR-Vβ^+^ expansions in T-LGLL patients expressed significantly higher levels of CD57 and 2B4 and significantly lower levels of CD27 and CD127 compared with the dominant CD8^+^ TCR-Vβ^+^ expansions in dasatinib-treated CML patients (Fig. 3B, top panel). However, no significant phenotypic differences were observed between the residual CD8^+^ TCR-Vβ^−^ populations in T-LGLL patients and the residual CD8^+^ TCR-Vβ^−^ populations in dasatinib-treated CML patients (Fig. 3B, bottom panel).

### Phenotypic parallels between CMV-specific CD8^+^ T-cells and dominant CD8^+^ TCR-Vβ^+^ expansions in T-LGLL patients

**Figure 4:**
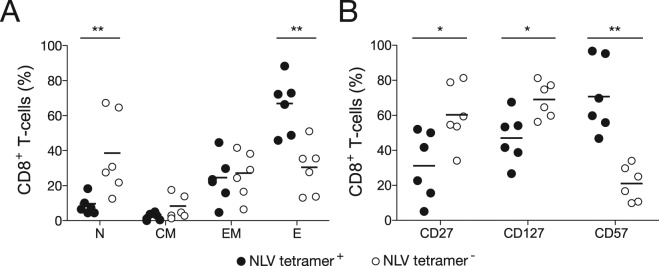
Phenotypic analysis of CMV-specific CD8^+^ T-cells in healthy donors. (**A**) Percent frequencies of NLV/HLA-A*0201 tetramer^+^ (black dots) and NLV/HLA-A*0201 tetramer^−^ CD8^+^ T-cells (white dots) displaying the indicated differentiation phenotypes in healthy donors (n = 6). (**B**) Percent frequencies of NLV/HLA-A*0201 tetramer^+^ (black dots) and NLV/HLA-A*0201 tetramer^−^ CD8^+^ T-cells (white dots) expressing the indicated markers in healthy donors (n = 6). Statistical comparisons were performed using the Mann-Whitney U test. **P < 0.001, *P < 0.01. Horizontal bars indicate mean values. N, naïve; CM, central-memory; EM, effector-memory; E, effector.

In further experiments, we evaluated the phenotypic characteristics of CMV pp65-specific CD8^+^ T-cells in healthy HLA-A2^+^ individuals (Fig. 4). Terminally differentiated (CCR7^−^ CD45-RA^+^) effector cells predominated in the NLV/HLA-A*0201 tetramer^+^ fraction (Fig. 4A), which also expressed low levels of CD27, intermediate levels of CD127, and high levels of CD57 (Fig. 4B). This effector-polarized differentiation profile closely resembles the phenotype of dominant CD8^+^ TCR-Vβ^+^ expansions in T-LGLL patients. In contrast, the NLV/HLA-A*0201 tetramer^–^ fraction incorporated higher frequencies of naïve and less differentiated memory cells, with a balanced EM/E profile reminiscent of the dominant CD8^+^ TCR-Vβ^+^ expansions in dasatinib-treated CML patients and the residual CD8^+^ TCR-Vβ^−^ populations in both T-LGLL and dasatinib-treated CML patients.

### Dominant CD8^+^ TCR-Vβ^+^ expansions in T-LGLL and dasatinib-treated CML patients are clonotypically distinct

**Figure 5:**
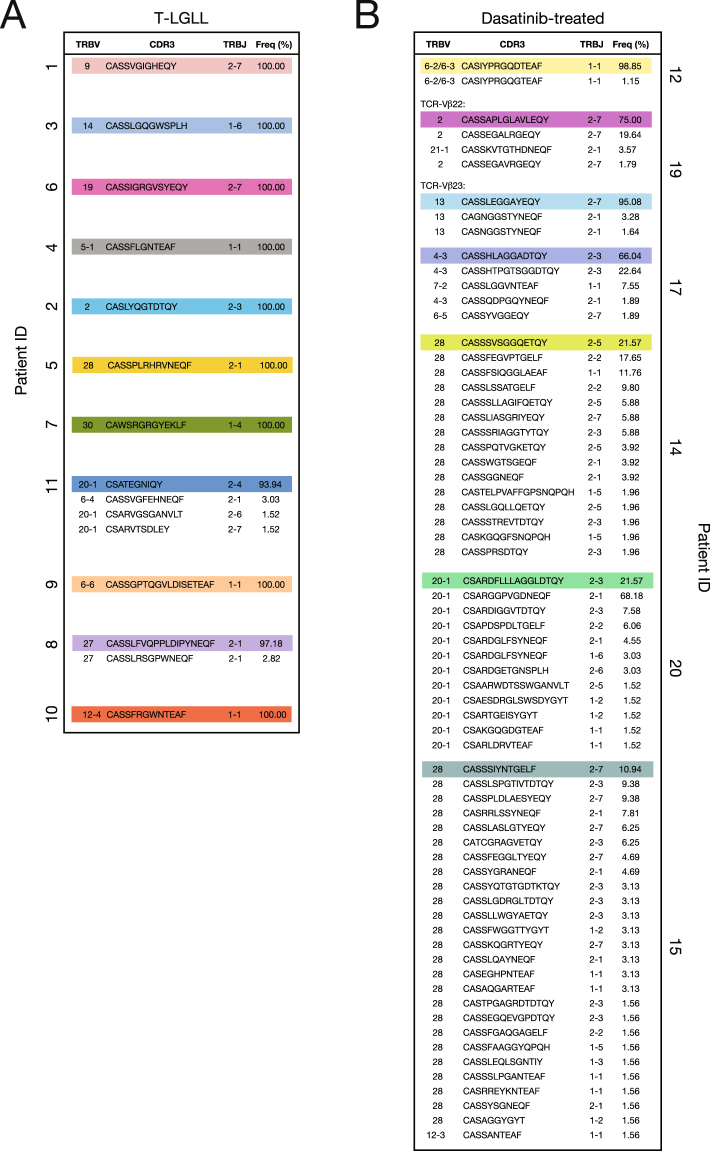
Clonotypic analysis of dominant CD8^+^ TCR-Vβ^+^ expansions in T-LGLL and dasatinib-treated CML patients. (**A**) Percent frequencies of expanded (TCR-Vβ^+^) CD8^+^ T-cell clonotypes in T-LGLL patients. (**B**) Percent frequencies of expanded (TCR-Vβ^+^) CD8^+^ T-cell clonotypes in dasatinib-treated CML patients. Gene usage and CDR3β amino acid sequences are listed, and dominant clonotypes are highlighted in color. Data are shown for two co-dominant CD8^+^ TCR-Vβ^+^ expansions in dasatinib-treated CML patient 19.

Next, we performed a molecular analysis of expressed *TRB* gene rearrangements in CD3^+^ CD8^+^ TCR-Vβ^+^ cell populations sorted directly *ex vivo* from T-LGLL and dasatinib-treated CML patients. The dominant CD8^+^ TCR-Vβ^+^ expansions in T-LGLL patients were largely monoclonal (Fig. 5A), whereas the dominant CD8^+^ TCR-Vβ^+^ expansions in dasatinib-treated CML patients were either oligoclonal or polyclonal (Fig. 5B). However, *in vitro* culture revealed the presence of additional clonotypes with identical *TRBV* gene-encoded segments in the dominant CD8^+^ TCR-Vβ^+^ expansions isolated from T-LGLL patients (Supplementary Figure 2). Although clonotypic drift is a recognized feature of dominant T-LGL populations *in vivo*^2^, the emergence of subdominant clonotypes *in vitro* likely reflects a proliferative advantage over more terminally differentiated and potentially senescent dominant clonotypes.

### Dasatinib administration rapidly increases lymphocyte numbers and further inflates dominant CD8^+^ TCR-Vβ^+^ expansions

**Figure 6:**
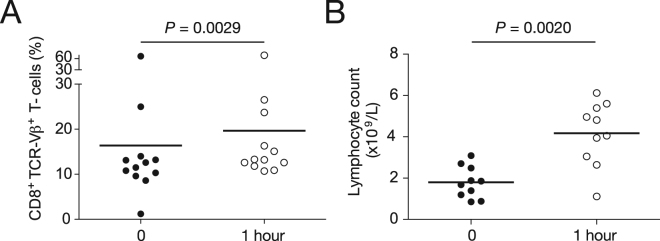
Influence of dasatinib on lymphocyte numbers and dominant CD8^+^ TCR-Vβ^+^ expansions in CML patients. (**A**) Percent frequencies of dominant CD8^+^ TCR-Vβ^+^ expansions in CML patients before and 1 hour after a single 100 mg dose of dasatinib. (**B**) Total lymphocyte counts in CML patients before and 1 hour after a single 100 mg dose of dasatinib. Statistical comparisons were performed using the Wilcoxon matched pairs test. Horizontal bars indicate mean values.

To examine the impact of dasatinib on the CD8^+^ T-cell repertoire in CML patients, we administered 100 mg of the drug and compared blood samples drawn at baseline with blood samples drawn after 1 hour. The percent frequencies of the dominant CD8^+^ TCR-Vβ^+^ expansions increased significantly over this brief time period (Fig. 6A). However, the corresponding total lymphocyte counts also increased significantly (Fig. 6B), indicating that the effects of dasatinib were not confined to the dominant CD8^+^ TCR-Vβ^+^ expansions^29^.

## Discussion

In this study, we performed a comprehensive phenotypic and clonotypic assessment of expanded (TCR-Vβ^+^) and residual (TCR-Vβ^−^) CD8^+^ T-cell populations in T-LGLL and dasatinib-treated CML patients. The dominant CD8^+^ TCR-Vβ^+^ expansions in T-LGLL patients were significantly larger and more differentiated than the dominant CD8^+^ TCR-Vβ^+^ expansions in dasatinib-treated CML patients. Molecular analysis of expressed *TRB* gene rearrangements further showed that the dominant CD8^+^ TCR-Vβ^+^ expansions in T-LGLL patients were largely monoclonal, whereas the dominant CD8^+^ TCR-Vβ^+^ expansions in dasatinib-treated CML patients were either oligoclonal or polyclonal. These distinct features suggest that key mechanistic differences underlie the genesis and maintenance of dominant CD8^+^ TCR-Vβ^+^ expansions in T-LGLL and dasatinib-treated CML patients.

In conjunction with a terminally differentiated (CCR7^−^ CD45-RA^+^) effector phenotype, the dominant CD8^+^ TCR-Vβ^+^ expansions in T-LGLL patients expressed high levels of CD57, which is thought to indicate replicative senescence as a consequence of antigen-driven proliferation^30^, and correspondingly low levels of immunoregulatory markers, such as PD-1^31^. These findings suggest a central role for persistent antigens in the pathogenesis of T-LGLL. In line with this interpretation, we found strong phenotypic parallels between CMV pp65-specific CD8^+^ T-cells in healthy donors and the dominant CD8^+^ TCR-Vβ^+^ expansions in T-LGLL patients. Moreover, clonal CD8^+^ CD57^+^ cells in T-LGLL patients can be derived from CD8^+^ CD57^–^ progenitors *in vitro*, suggesting that an earlier memory compartment sustains the dominant CD8^+^ TCR-Vβ^+^ expansions *in vivo*^18^.

STAT3 is a latent transcription factor and a key component of the JAK/STAT signalling pathway^32^, linked in a dysregulated state with tumor growth and pro-oncogenic inflammation^33^. Indeed, persistent activation of STAT3 has been observed in at least 22 different cancers^34^, including T-LGLL^21,35^. Genomic studies have revealed that up to 40% of T-LGLL patients harbour STAT mutations^26^. Although the majority of these somatic variants localize to STAT3^26^, comparable mutations have been observed in STAT5B^28^. Other signalling pathways can also impact the function of STAT3^21,36^. On the basis of these observations, it has been proposed that STAT mutations drive the monoclonal CD8^+^ TCR-Vβ^+^ expansions that characterize T-LGLL. However, primary T-LGLs are by no means immortal *in vitro* like many classical tumor cell lines^37,38^. It therefore seems more likely that STAT mutations accumulate as a consequence of prolonged antigenic stimulation within a homeostatically conducive environment^39,40^.

In dasatinib-treated CML patients, the expanded (TCR-Vβ^+^) and residual (TCR-Vβ^−^) CD8^+^ T-cell populations were phenotypically similar and resembled healthy memory CD8^+^ T-cells. Moreover, the dominant CD8^+^ TCR-Vβ^+^ expansions were either oligoclonal or polyclonal, which may reflect either multiple antigen specificities or germline-encoded bias within a single antigen specificity^41^. Although the actions of dasatinib are poorly understood, it is possible that the short half-life of the drug *in vivo* leads to diurnal cycles of systemic immunosuppression^11,42^. It has also been shown that naïve T-cells are more susceptible to the effects of dasatinib than memory T-cells^43^. Dasatinib therapy may therefore create an immunosuppressive environment that favors the expansion of pre-existing memory CD8^+^ T-cells, potentially mimicking the conditions encountered after allogeneic stem cell transplantation^7^ or during primary infection with certain persistent viruses^44,45^. This mechanistic interpretation is consistent with the absence of STAT mutations in dasatinib-associated CD8^+^ TCR-Vβ^+^ expansions (Table 1).

**Figure 7:**
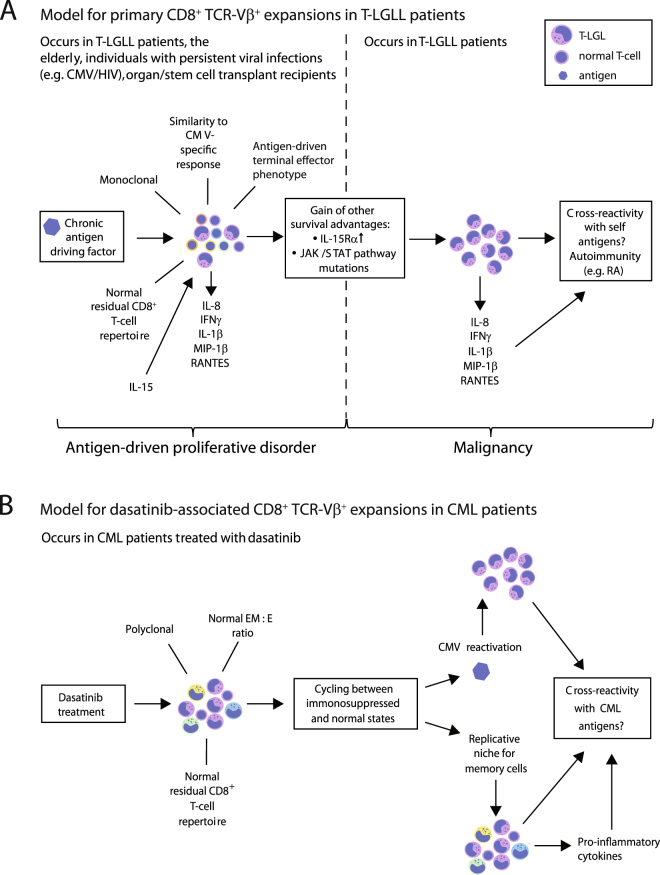
Immunopathogenesis of dominant CD8^+^ TCR-Vβ^+^ expansions in T-LGLL and dasatinib-treated CML patients. (**A**) Hypothetical model showing the potential mechanisms that drive CD8^+^ TCR-Vβ^+^ expansions in T-LGLL patients. In this model, persistent antigenic drive and homeostatic cytokines facilitate the expansion of highly responsive clonotypes, which subsequently acquire somatic mutations that confer additional proliferative and/or survival advantages. These selection events culminate in the outgrowth of a dominant CD8^+^ TCR-Vβ^+^ population. Autoimmunity may be triggered via the release of pro-inflammatory mediators and/or self-antigen recognition via the expressed TCR. (**B**) Hypothetical model showing the potential mechanisms that drive CD8^+^ TCR-Vβ^+^ expansions in dasatinib-treated CML patients. In this model, the short half-life of dasatinib *in vivo* leads to diurnal cycles of systemic immunosuppression, which may provide a replicative niche for memory T-cells and/or promote the reactivation of persistent viruses, such as CMV. These effects drive polyclonal CD8^+^ TCR-Vβ^+^ expansions. Improved disease outcomes may arise as a consequence of CML-associated antigen cross-recognition via the expressed TCRs. EM, effector-memory; E, effector.

Collectively, our data support the view that monoclonal CD8^+^ TCR-Vβ^+^ expansions in T-LGLL patients arise in response to chronic antigen-driven stimulation and then acquire somatic mutations that provide further survival advantages (Fig. 7A). In contrast, the dominant CD8^+^ TCR-Vβ^+^ expansions in dasatinib-treated CML patients appear to be more benign and most likely originate from generic perturbations that affect the pre-existing pool of memory CD8^+^ T-cells (Fig. 7B). Further studies are therefore required to identify the antigenic culprits that trigger T-LGLL and characterize the dominant CD8^+^ TCR-Vβ^+^ expansions that potentially cause autoimmune side-effects and/or beneficially target leukemic cells in dasatinib-treated CML patients^46^.

## Materials and Methods

### Study subjects and samples

PBMCs used for the characterization of chronic viral antigen-specific CD8^+^ T-cell populations were isolated from the blood of healthy HLA-A2^+^ CMV^+^ individuals (age range, 24–72 years) via standard lymphocyte purification protocols. Equivalent samples from T-LGLL and dasatinib-treated CML patients were obtained with approval from the relevant local Ethics Committees and Institutional Review Boards. All subjects provided written informed consent in accordance with the Declaration of Helsinki. PBMCs from patients 7–11 were collected under protocols approved by the Institutional Review Board of the National Heart, Lung, and Blood Institute, National Institutes of Health, Bethesda MD, USA. All other patient samples were obtained from Helsinki University Hospital under protocols approved by The Hospital District of Helsinki and Uusimaa Medicinal Ethical Committee, Finland. Clinical details are summarized in Table 1 and Supplementary Table 1. The diagnosis of T-LGLL was based on the following WHO criteria: a monoclonal TCR rearrangement, the presence of an abnormal cytotoxic T-cell population with expression of CD3, CD8, and CD57 detected by flow cytometry, and a peripheral blood smear LGL count of >2 × 10^9^/L. If all other criteria were met, a diagnosis of T-LGLL was also considered with a peripheral blood smear LGL count of <2 × 10^9^/L. Screening for STAT3 and STAT5B mutations was performed via amplicon sequencing across the hotspot area^47^.

### Flow cytometry reagents

PE-conjugated HLA-A2 tetramers refolded around the CMV pp65_495-503_ epitope NLVPMVATV (NLV) were generated as described previously^48,49^ and used at a final concentration of 10 μg/mL. Directly conjugated mAbs were obtained from commercial suppliers: (i) anti-CD3-APC-H7, anti-CD8-BV786, anti-CD14-BV510, anti-CD19-BV510, anti-CD45-RA-PerCP-Cy5.5, anti-CD57-APC, anti-CD127-BV421, anti-CD160-PerCP-Cy5.5, anti-CCR7-PE-Cy7, anti-CTLA-4-PE-Cy7, anti-BTLA-APC, anti-2B4-FITC, anti-2B4-PE, and anti-Tim-3-AF700 (BD Biosciences); (ii) anti-CD27-BV605 and anti-PD-1-PE/Dazzle 594 (BioLegend); and (iii) a panel of anti-TCR-Vβ-FITC and anti-TCR-Vβ-PE mAbs (Beckman Coulter). The acute effects of dasatinib administration were monitored using an IOTest Beta Mark TCR Repertoire Kit (BD Biosciences). Non-viable cells were excluded from the analysis using LIVE/DEAD Fixable Aqua (Life Technologies). Data were acquired using a Fortessa X-20 flow cytometer (BD Biosciences) and analyzed with FlowJo software version 10.0.7 (Tree Star). Viable CD3^+^ CD8^+^ TCR-Vβ^+^ cells were sorted using an Influx^TM^ Cell Sorter (BD Biosciences).

### Clonotypic analysis

Molecular analysis of expressed *TRB* gene rearrangements in flow-sorted CD3^+^ CD8^+^ TCR-Vβ^+^ cell populations was performed using a template-switch anchored RT-PCR^50^. Gene usage was assigned via web-based alignment of molecular transcripts in accordance with the ImMunoGeneTics (IMGT) information system (http://www.imgt.org).

### Cell culture

Flow-sorted CD3^+^ CD8^+^ TCR-Vβ^+^ cells were stimulated non-specifically with 1 μg/mL phytohemagglutinin (Fisher Scientific) in the presence of irradiated PBMCs from three healthy donors. Cultures were maintained in RPMI 1640 medium containing 100 U/mL penicillin, 100 mg/mL streptomycin, 2 mM L-glutamine, 10% heat-inactivated fetal bovine serum, and 5% heat-inactivated human AB serum (all from Life Technologies), supplemented with 200 IU/mL interleukin (IL)-2 and 25 ng/mL IL-15 (PeproTech).

### Statistical analysis

Univariate statistical analyses were implemented using Prism 5 software (GraphPad). Comparisons between groups were performed using either the Mann-Whitney U test or the Wilcoxon matched pairs test. *P* values < 0.05 were considered significant.

### Data availability

The data that support the findings of this study are available from the corresponding author on reasonable request.

## Electronic supplementary material

Supplementary Information
