# Supplementary material for: Divergent roles for antigenic drive in the aetiology of primary versus dasatinib-associated CD8+ TCR-Vβ+ expansions
Source: Sci Rep. 2018 Feb 7;8:2534. doi: 10.1038/s41598-017-18062-x (PMC5803196; doi:10.1038/s41598-017-18062-x)
Supplement: Supplementary file 1 — Supplementary Information [file 41598_2017_18062_MOESM1_ESM.pdf]

SUPPLEMENTARY INFORMATION

Divergent roles for antigenic drive in the aetiology of primary versus dasatinib-associated CD8$^+$ TCR-Vβ^+ expansions

Anna Lissina[1]*, James E. McLaren[2]*, Mette Ilander[3], Emma I. Andersson[3], Catherine S. Lewis[1], Mathew Clement[2], Andrew Herman[1], Kristin Ladell[2], Sian Llewellyn-Lacey[2], Kelly L. Miners[2], Emma Gostick[2], J. Joseph Melenhorst[4], A. John Barrett[4], David A. Price[2]*, Satu Mustjoki[3]*, Linda Wooldridge[1]*

[1]Faculty of Health Sciences, University of Bristol, Biomedical Sciences Building, Bristol, UK

[2]Institute of Infection and Immunity, Cardiff University School of Medicine, Heath Park, Cardiff, UK

[3]Hematology Research Unit Helsinki, Department of Clinical Chemistry and Hematology, University of Helsinki and Helsinki University Hospital Comprehensive Cancer Center, Helsinki, Finland

[4]Stem Cell Allogeneic Transplantation Section, Hematology Branch, National Heart, Lung, and Blood Institute, National Institutes of Health, Bethesda, Maryland, USA

*These authors contributed equally to this manuscript.

Correspondence: Dr Anna Lissina, Faculty of Health Sciences, University of Bristol, Biomedical Sciences Building, University Walk, Bristol BS8 1TD, UK.

E-mail: a.lissina@bristol.ac.uk

Supplementary Figure Legends

Supplementary Figure 1. Identification of dominant $CD8^+$ TCR-$V\beta^+$ expansions in T-LGLL and dasatinib-treated CML patients. Representative anti-TCR-Vβ screening profiles are shown for the indicated T-LGLL (left panel) and dastinib-treated CML patients (right panel).

Supplementary Figure 2. Clonotypic analysis of dominant $CD8^+$ TCR-$V\beta^+$ expansions in T-LGLL patients. (**A**) Percent frequencies of expanded (TCR-$V\beta^+$) $CD8^+$ T-cell clonotypes sorted directly *ex vivo* from T-LGLL patients. (**B**) Percent frequencies of expanded (TCR-$V\beta^+$) $CD8^+$ T-cell clonotypes restimulated twice *in vitro* over a period of 1 month after sorting from T-LGLL patients. Gene usage and CDR3β amino acid sequences are listed, and dominant *ex vivo* clonotypes are highlighted in color.

Supplementary Figure 1

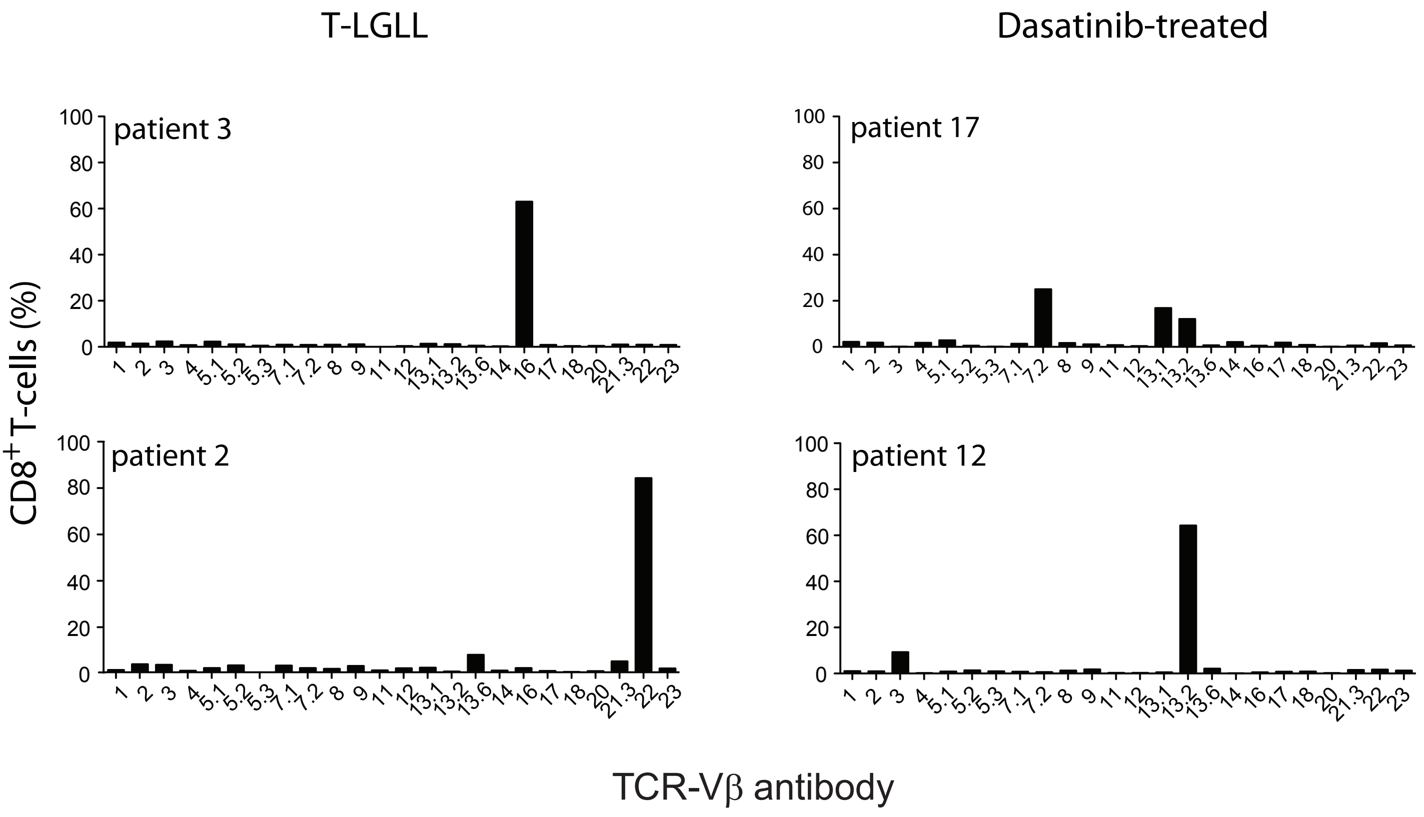

Supplementary Figure 2

A

Ex vivo

Patient ID 1

TRBV	CDR3β	TRBJ	Freq (%)
9	CASSVGIGHEQY	2-7	100.00

Patient ID 3

TRBV	CDR3β	TRBJ	Freq (%)
14	CASSLGQGWSPLH	1-6	100.00

B

After *in vitro* culture

Patient ID 1

TRBV	CDR3β	TRBJ	Freq (%)
9	CASSVHSSTYEQY	2-7	25.00
9	CASSEGTSDTDTQY	2-3	14.71
9	CASSVEGYNEQF	2-1	13.24
9	CASSISGSGEQF	2-1	11.76
9	CASSTSTGTNQPQH	1-5	10.29
9	CASSVDTSNQPQH	1-5	7.35
9	CASSTSGQALEAF	1-1	7.35
9	CASTGGLNYGYT	1-2	4.41
9	CASSVGQGQETQY	2-5	2.94
9	CASSVGQGQETQY	2-5	1.47
9	CASSGYSNQETQY	2-5	1.47

Patient ID 3

TRBV	CDR3β	TRBJ	Freq (%)
14	CASSQDLGTRFEVQY	2-4	64.20
14	CASSQAPRNPNYGYT	1-2	13.58
14	CASSQGFNEQF	2-1	9.88
14	CASSQTSRRLNEQF	2-1	3.70
14	CASSHPRNPGGGNTGELF	2-2	2.47
14	CASSLESDTDTQY	2-3	2.47
14	CASSQVGDEQF	2-1	2.47
14	CASSPGRTDTQY	2-3	1.23

Patient ID	Leukemia type	Dominant TCR-Vβ expansion (Arden)	$CD8^+$ TCR-$V\beta^+$ T-cells (%) before dasatinib	$CD8^+$ TCR-$V\beta^+$ T-cells (%) 1 hour post-dasatinib	Lymphocyte count ($x10^9$/L) before dasatinib	Lymphocyte count ($x10^9$/L) 1 hour post-dasatinib	CMV serostatus
12	CML	13.2	66.4	69.2	0.88	2.64	Positive
13	CML	14	14	13.3	1.39	3.94	Positive
14	CML	3	10.3	10.7	3.09	4.96	Positive
15	CML	3	11.5	13.2	1.68	5.39	Positive
16	CML	5.3	1.2	23.7	0.85	0.78	Negative
17	CML	7.2	25	26.5	2.49	4.81	Positive
18	CML	2	13.1	15.1	1.88	6.12	NA
18	CML	8	12.6	12.6	1.88	6.12	NA
19	CML	22	10.8	12.6	1.85	3.05	Negative
19	CML	23	9.6	10.9	1.85	3.05	Negative
20	CML	2	8.6	11.8	2.7	5.6	Negative
21	CML	14	11.6	14.1	1.19	4.05	Positive

Supplementary Table 1A: Additional clinical details of dasatinib-treated CML patients. NA: not available.

Patient ID	Leukemia type	Time of sampling relative to dasatinib administration	CMV IgG (aU/mL)	CMV serostatus
12	CML	-	107	Positive
13	CML	0 hours (before dasatinib)	44	Positive
14	CML	2 hours post-dasatinib	70	Positive
15	CML	1 hour post-dasatinib	102	Positive
16	CML	1 hour post-dasatinib	0	Negative
17	CML	NA	72	Positive
18	CML	2 hours post-dasatinib	NA	NA
19	CML	1 hour post-dasatinib	<4	Negative
20	CML	1 hour post-dasatinib	<4	Negative
21	CML	NA	>250	Positive

Supplementary Table 1B: Time of sampling relative to dasatinib administration for patients shown in Supplementary Table 1A. NA: not available.
